# Volumetric and Functional Features of Left Atrium in Chronic Schizophrenia—Detailed Analysis from Three-Dimensional Speckle-Tracking Echocardiographic MAGYAR-Path Study

**DOI:** 10.3390/biomedicines14051088

**Published:** 2026-05-12

**Authors:** Attila Nemes, Renáta Halcsik, Árpád Kormányos, Nándor Gyenes, Asghar Keifari, Bence András Lázár, Csaba Lengyel, János Kálmán

**Affiliations:** 1 Department of Medicine, Albert Szent-Györgyi Medical School, University of Szeged, Semmelweis Street 8, P.O. Box 427, H-6725 Szeged, Hungary; halcsik.renata@med.u-szeged.hu (R.H.); kormanyos.arpad@med.u-szeged.hu (Á.K.); gyenes.nandor@med.u-szeged.hu (N.G.); lengyel.csaba@med.u-szeged.hu (C.L.); 2 Department of Psychiatry, Albert Szent-Györgyi Medical School, University of Szeged, H-6720 Szeged, Hungary; asghar.keifari@med.u-szeged.hu (A.K.); lazar.bence.andras@med.u-szeged.hu (B.A.L.); kalman.janos@med.u-szeged.hu (J.K.)

**Keywords:** schizophrenia, chronic, cardiovascular disease, three-dimensional, speckle-tracking, echocardiography, left atrium, strain, volume

## Abstract

**Introduction**: Health problems related to cardiovascular morbidity and mortality are overrepresented in patients with schizophrenia (SCH) and their rates have not declined in parallel with those of the general population. Cardiovascular diseases in patients with SCH are less likely to be diagnosed and treated, and data regarding structural and functional cardiac abnormalities—particularly those involving the left atrium (LA)—remain limited in this population. The present study is the first to provide a detailed three-dimensional speckle-tracking echocardiography (3DSTE)-derived volumetric and functional evaluation of LA properties in patients with chronic SCH compared with age-, gender- and body mass index (BMI)-matched healthy controls (HCs). **Methods**: A total of 36 patients with SCH were initially enrolled, from which 19 subjects (53%) were excluded due to inferior image quality. Ultimately, 17 SCH patients (mean age: 45.2 ± 7.7 years; 9 males, 53%) were compared with 40 age- and gender-matched HCs (mean age: 42.5 ± 5.7 years; 23 males, 58%). All participants underwent comprehensive two-dimensional Doppler echocardiography and 3DSTE. **Results**: LA volumes respecting the cardiac cycle were lower in SCH patients compared with controls. Among LA volume-derived functional properties, total and active LA stroke volumes were reduced in patients with chronic SCH, whereas passive LA emptying fraction was increased. All global and mean segmental peak LA strain parameters tended to be increased in SCH patients, with global and mean segmental LA area strain (AS) and mean segmental LA radial strain (RS) reaching statistical significance. Regarding regional peak LA strains, basal LA circumferential strain (CS) and LA-AS, as well as superior LA longitudinal strain (LS), LA-CS, and LA-AS, differed significantly between the groups. All global and mean segmental LA strain parameters measured at atrial contraction tended to be increased in chronic SCH patients, with global and mean segmental LA-AS and mean segmental LA-RS and LA-LS reaching statistical significance. Regional LA strains during atrial contraction demonstrated increased superior LA-RS, LA-CS, LA-LS and LA-AS, along with elevated mid-atrial LA-RS, LA-AS and LA-3D strain. All these abnormalities suggest reduced LA volumes in all phases of LA function, accompanied by overcompensating functional alterations. **Conclusions**: Chronic schizophrenia is associated with marked volumetric and functional abnormalities of the left atrium. These findings highlight the need for comprehensive cardiac functional evaluation extending beyond left ventricular-centered analysis in patients with this severe mental illness.

## 1. Introduction

Cardiovascular diseases (CVDs) represent the most frequent comorbid somatic conditions in patients with severe mental disorders such as schizophrenia (SCH), contributing to increased mortality and reduced life expectancy [1,2]. Unfortunately, several challenges remain in the screening, recognition and proper treatment of CVDs in patients with chronic SCH [3]. From the patients’ perspective, SCH-related psychopathology, such as partial or complete lack of insight and limited cooperation, are the major inhibitors of holistic diagnostic and therapeutic approaches. In addition, basic and normative data about structural and functional cardiac pathologies are sparse or missing in this population [4]. Most existing studies have focused on left ventricular (LV) myocardial mechanics and have reported LV strain abnormalities regardless of the imaging modality used [5,6,7]. Other reports have associated reduced LV ejection fraction with the negative impact of long-term use of antipsychotic medications during lifelong treatment of chronic SCH [8].

To date, however, the mechanical properties of the left atrium (LA)—the other key left-sided component of the systemic circulation—have remained unexplored in patients with chronic SCH. The LA plays a central role in maintaining the circulation through its complex functional properties [9,10,11,12,13]. One of the most advanced cardiovascular imaging techniques, three-dimensional (3D) speckle-tracking echocardiography (3DSTE), allows for detailed evaluation of the LA volumetric changes respecting the cardiac cycle, as well as characterization of LA functional properties using both volume-based and strain parameters [14,15,16,17,18,19,20]. Previous studies have suggested that certain pathological conditions may be associated with specific LA abnormalities [20]. The present study is the first to provide a detailed 3DSTE-derived volumetric and functional evaluation of LA properties in patients with chronic SCH.

## 2. Materials and Methods

### 2.1. Patient Population

The study initially included 36 patients with chronic SCH, of whom 19 (53%) were excluded due to inferior image quality. The final study population consisted of 17 patients (mean age: 45.2 ± 7.7 years; 9 males, 53%). Patients under the age of 18 and over the age of 65 were not eligible for inclusion. SCH subjects were pre-screened and selected by a trained psychiatrist. The diagnosis of chronic SCH (multiple episodes, currently in remission) was established according to ICD-11 code 6A20.11 criteria based on a review of the medical history and an interview, including evaluation of the current clinical picture, collecting information from the clinical staff and caregivers [21]. Patients in the acute phase of psychosis were not enrolled in the study. Verified medical records confirming a history of more than 5 psychotic episodes were required for inclusion. Mental and somatic medical history, pharmacological treatment history, smoking habits and current medications were collected. The presence of CVD risk factors was not considered an exclusion criterion. Results were compared with those of 40 age- and gender-matched healthy controls (HCs) (mean age: 42.5 ± 5.7 years; 23 males, 58%). All SCH patients and HCs voluntarily agreed to participate in the present study. Patients with SCH were treated and followed at the Department of Psychiatry, University of Szeged, Hungary, a tertiary referral center. Baseline somatic parameters, including body weight (88.9 ± 12.8 kg vs. 86.3 ± 10.0 kg, *p* = ns), height (170.0 ± 9.8 cm vs. 171.1 ± 6.5 cm, *p* = ns), body surface area (2.04 ± 0.17 m^2^ vs. 2.00 ± 0.15 m^2^, *p* = ns) and body mass index (31.1 ± 5.6 kg/m^2^ vs. 30.2 ± 4.2 kg/m^2^, *p* = ns) did not differ significantly between the groups. Healthy subjects underwent laboratory testing, physical examination, standard 12-lead electrocardiography (ECG), and two-dimensional (2D) Doppler echocardiography, and all parameters were found to be within the normal reference ranges. None of the healthy subjects had a known disorder or pathological state, were receiving regular medication, were smokers, or were professional athletes. Routine two-dimensional (2D) Doppler echocardiography extended with 3DSTE was performed for all participants. The present study is part of a large-scale clinical study organized at our department called the **M**otion **A**nalysis of the heart and **G**reat vessels b**Y** three-dimension**A**l speckle-t**R**acking echocardiography in **Path**ological cases (**MAGYAR-Path**) **Study**, which aims to analyze the diagnostic and prognostic value of 3DSTE-derived parameters in certain disorders like schizophrenia (‘magyar’ means ‘Hungarian’ in the Hungarian language) [18,19,20]. All participants gave written informed consent. The study protocol was approved by the Institutional and Regional Human Biomedical Research Committee of the University of Szeged, Hungary (No.: 71/2011, latest approval 17 March 2025) and by the Scientific and Research Ethics Committee with latest extension approval for CS-SCH populations (IV/1230-6/2022/EKU). The study complied with the ethical guidelines of the Declaration of Helsinki. All healthy subjects and patients gave informed consent.

### 2.2. Two-Dimensional Doppler Echocardiography

All participants underwent 2D Doppler echocardiography using a Toshiba Artida cardiac ultrasound machine (Toshiba Medical Systems, Tokyo, Japan) equipped with a broadband 1–5 MHz PST-30BT phased-array transducer. International guidelines were followed during chamber quantifications [22]. Significant valvular regurgitation and stenosis were excluded by Doppler echocardiography.

### 2.3. Three-Dimensional Speckle-Tracking Echocardiography

3DSTE examinations were performed in two steps [18,19]. Immediately after the 2D echocardiographic study, 3D echocardiographic datasets (‘echocloud’) were acquired using the same Toshiba Artida echocardiographic tool, after switching to a fully sampled PST-25SX matrix-array transducer (Toshiba Medical Systems, Tokyo, Japan). Then six wedge-shaped subvolumes were acquired from the apical window during a single breath-hold over consecutive cardiac cycles while maintaining a constant RR interval on the ECG. Volumetric and functional analysis of the LA was performed at a later date using the acquired ‘echocloud’ and vendor-derived 3D Wall Motion Tracking software version 2.5 (Toshiba Medical Systems, Tokyo, Japan). Apical four- and two-chamber long-axis views and short-axis views at basal, mid-atrial, and superior regional LA levels were automatically selected from the datasets. In long-axis views, the border of the LA was manually traced by placing multiple reference points from the lateral LV-mitral annular edge through the LA apex to the septal LV-mitral annular edge. The LA appendage and the pulmonary veins were not considered to be part of the LA. At end-diastole, the contour of the LA was detected and the 3D endocardial surface was reconstructed and tracked in the 3D space during the cardiac cycle, resulting in a 3D virtual LA cast. As a result, the following LA volumes were measured [18]:

- V_max_ = maximum LA volume (the largest LA volume at end-systole, immediately before mitral valve opening);
- V_min_ = minimum LA volume (the smallest LA volume at end-diastole, immediately before mitral valve closure);
- V_preA_ = LA volume before atrial contraction (the last frame before mitral valve reopening or at the onset of the P wave on the ECG at early diastole).

Using these LA volumes, several LA volume-based functional properties were measured (see Table 1). At the same time, using the same 3D LA cast, the following LA strain parameters were also measured [19]:

Unidimensional/unidirectional LA strains:

- Radial strain (RS) representing myocardial wall thinning and thickening.
- Longitudinal strain (LS) representing myocardial wall lengthening and shortening.
- Circumferential strain (CS) representing myocardial wall widening and narrowing.

Multidimensional/multidirectional complex LA strains:

- Area strain (AS), which is a combination of LS and CS.
- 3D strain (3DS), which is a combination of RS, LS and CS.

**Figure 1:**
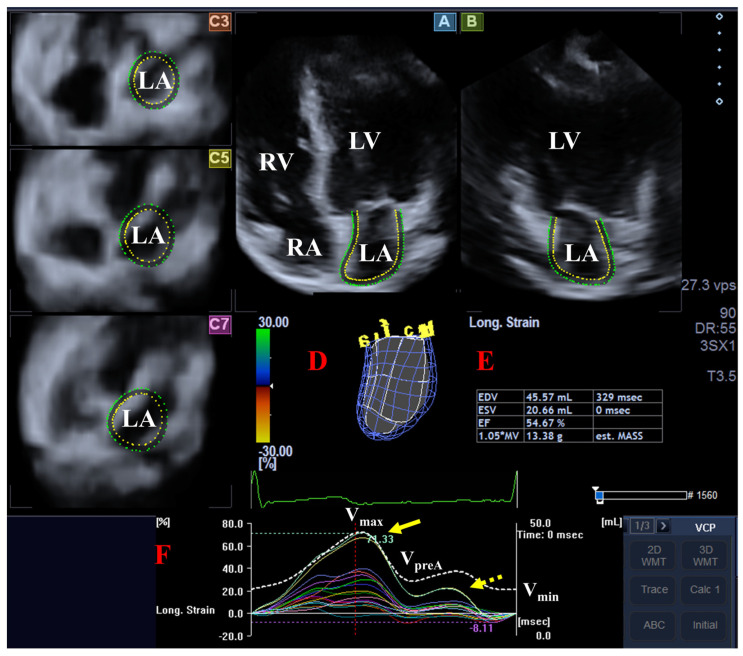
Three-dimensional speckle-tracking echocardiographic left atrial (LA) analysis is presented: (**A**) apical four-chamber and (**B**) two-chamber views, and short-axis views (**C3**) at the basal, (**C5**) mid-atrial and (**C7**) superior LA levels are demonstrated (**D**) with a three-dimensional LA cast. (**E**) Calculated volumetric LA data are demonstrated together with (**F**) time—global LA volume change (dashed line) and time—segmental LA longitudinal strain curves (colored lines). The yellow arrow represents peak LA strains, while the yellow dotted arrow represents LA strains at atrial contraction. Abbreviations: EDV = end-diastolic volume, ESV = end-systolic volume, EF = ejection fraction, LA = left atrium, LV = left ventricle, RA = right atrium, RV = right ventricle, V_max_ = maximum LA volume, V_preA_ = pre-atrial contraction LA volume, and V_min_ = minimum LA volume.

LA strain parameters representing the end-systolic reservoir phase (peak LA strain) and late-diastolic active contraction (LA strain during atrial contraction) phase of the LA function were determined. Segmental, regional, mean segmental and global LA strains were measured using the typical twin-peak LA strain curve (Figure 1). The segmentation model created for LV analysis was used for the evaluation of the LA.

### 2.4. Statistical Analysis

Continuous variables are presented as mean ± standard deviation, whereas categorical variables are expressed as *n* (%). Student’s *t* test, Χ^2^ test, and Fisher’s exact tests were used for group comparisons. A 2-tailed *p* < 0.05 was considered to be statistically significant. Statistical power was retrospectively calculated using G*Power 3.1, based on the final sample size, a significance level of 0.05, and the observed effect size. Statistical analyses were performed using SPSS software (SPSS Inc., version 22, Chicago, IL, USA).

## 3. Results

### 3.1. Clinical and Demographic Data

None of the controls used any regular medication. Among patients with chronic SCH, beta-blocker therapy was used in one case, angiotensin-converting enzyme inhibitors in two cases, calcium-channel blockers in two cases, diuretics in two cases, and statins in two cases. None of the participants received antidiabetic therapy. From classical risk factors, diabetes mellitus was present in the medical history in two cases, hypertension in five cases and hyperlipidemia in seven cases. Antipsychotic treatment was used in all SCH patients.

### 3.2. Two-Dimensional Doppler Echocardiography

Routine 2D Doppler echocardiographic parameters showed no differences between patients with chronic SCH patients and matched HCs (Table 2). None of the participants showed ≥ grade 1 valvular regurgitation or any valvular stenosis.

### 3.3. 3DSTE-Derived LA Analysis

LA volumes respecting cardiac cycle were lower in patients with chronic SCH compared with controls. Among LA volume-derived functional parameters, LA-TASV and LA-AASV were lower in patients with chronic SCH, accompanied by increased LA-PAEF. All other SVs and EFs did not differ between the examined groups (Table 3). All global and mean segmental peak LA strain parameters showed a tendency toward higher values in patients with chronic SCH. Among these, global and mean segmental LA-AS and mean segmental LA-RS reached statistical significance (Table 4). From regional peak LA strain parameters, basal LA-CS and LA-AS, as well as superior LACS, LA-LS and LA-AS, differed significantly between the groups (Table 5). All global and mean segmental LA strains measured at atrial contraction tended to be higher in patients with SCH. Among these parameters, global and mean segmental LA-AS and mean segmental LA-RS and LA-LS reached statistical significance (Table 6). From regional LA strain parameters at atrial contraction, superior LA-RS, LA-CS, LA-LS and LA-AS were increased, as well as mid-atrial LA-RS, LA-3DS and LA-AS (Table 7). No associations were observed in patients with SCH between the severity of LA abnormalities and body mass index or the use of antipsychotic medications.

### 3.4. Post Hoc Power Analysis

A post hoc power analysis was performed to evaluate the robustness of the observed difference. With the current sample sizes, the analysis revealed a large effect size (Cohen’s d = 0.8) and an achieved power of 0.63 at an alpha level of 0.05.

## 4. Discussion

Several guidelines and consensus statements issued jointly by international cardiology and psychiatry societies emphasize the importance of identifying and managing cardiovascular disease in patients with severe mental illnesses such as SCH [2,23,24]. Although advanced imaging techniques such as 3DSTE provide highly accurate tools for identifying minor alterations in myocardial mechanics, there remains a significant gap in the literature regarding cardiac involvement in schizophrenia [7,14,15,16,17,18,19,20]. To the best of our knowledge, the present study provides the first evidence of LA volumetric and deformation abnormalities in patients with chronic SCH.

The LA undergoes dynamic volumetric and functional changes in coordination with the LV throughout the cardiac cycle [9,10,11,12,13]. During ventricular systole, the LA functions as a reservoir, reaching its maximal volume at end-systole. In early diastole, it serves as a passive conduit, facilitating blood flow from the pulmonary veins into the LV through the open mitral valve. Finally, during late diastole, the LA acts as an active booster pump, contracting to optimize LV filling and reaching its minimum volume just before mitral valve closure. Some clinical findings suggest that specific LA abnormalities may occur in certain disorders [20]. However, due to the lack of clinical data regarding LA volumetric and functional changes in SCH, to the best of the authors’ knowledge, this is the first study to assess these abnormalities in a series of chronic SCH patients.

The findings of the present study have several implications. First, 3DSTE proved to be a useful tool for the volumetric and functional assessment of the LA in patients with SCH. While these findings have been established in other patient cohorts within the MAGYAR-Path Study, evaluating this particular patient population presented greater technical and clinical challenges than those encountered in previous investigations. The behavioral characteristics of these patients and the need for specific handling during examinations may complicate image acquisition. In addition, body composition, smoking habits, and other lifestyle factors may contribute to the suboptimal image quality. These facts may partly explain the relatively low feasibility rate. Second, our results demonstrate that LA volumes are reduced in patients with chronic SCH compared with age-, gender- and BMI-matched healthy controls. Regarding reservoir function measured at end-systole, a reduced LA-SV was observed compared with controls, accompanied by a tendency towards increased LA thinning, lengthening and widening. These changes were represented by increased global and mean segmental peak LA strain parameters, with statistical significance observed for LA-RS and LA-AS. At a regional level, reduced basal LA widening (represented by basal LA-CS) and increased superior LA lengthening (represented by superior LA-LS) and widening (represented by superior LA-CS) were found. During early diastole, increased LA-PAEF was observed along with preserved LA-PASV. In late diastole, during the booster pump phase, reduced LA-AASV was associated with preserved LA-AAEF and tendentiously increased global and mean segmental LA strains, reaching statistical significance in the case of LA-AS. Increased basal LA-LS, mid-atrial LA-RS, LA-3DS and LA-AS; and superior LA-RS, LA-CS, LA-LS and LA-AS could also be presented. All these abnormalities suggested reduced LA volumes across all phases of LA function, accompanied by compensatory functional alterations in LA deformation parameters. The mechanism underlying these abnormalities remain uncertain. Reduced LA volumes can be a marker of LV dysfunction and heart failure. Moreover, the role of volume depletion and the effects of certain treatments cannot be ruled out either. The role of cardiovascular risk factors including hypertension, hyperlipidemia, diabetes, and smoking could not be excluded either.

Furthermore, the structural and functional LA abnormalities observed in patients with SCH may have a multifactorial etiology. This could involve genetic predisposition, as well as neurohormonal and immunological dysregulation of the heart–brain axis, leading to autonomic nervous system dysfunction [25,26,27]. Additionally, epigenetic factors—including metabolic cardiovascular risk factors—may interact with SCH-specific clinical components, such as lifestyle, mental and physical comorbidities, and issues with medication adherence and compliance [1,3]. Finally, the potential direct and indirect cardiotoxic effects of antipsychotics, particularly first- and second-generation agents, may further exacerbate cardiovascular risk in this population [4,8,28]. However, due to the cross-sectional pilot nature of the present study, the specific contribution of these factors cannot be determined. The authors wish to highlight that this research represents a novel area of inquiry; given the lack of prior literature, conclusions are inherently preliminary. These findings should be viewed as hypothesis-generating rather than definitive, reflecting the exploratory nature of this work. Moreover, further studies are required to clarify the cardiopathological mechanism underlying these observation in the LA. In addition, future clinical studies should evaluate the impact of antipsychotic therapy on LA structure and function, while also investigating whether early cardiac screening and intervention strategies could benefit this vulnerable population. Longitudinal follow-up of this cohort would provide crucial insights into the persistence of LA abnormalities over time and their potential correlation with long-term clinical outcomes.

## 5. Limitations Section

The present study has several limitations.

- First, the low number of patients with SCH represents an important limitation. If the number of cases had been larger, the statistical power of the analysis would have been greater. Although a larger number of patients were initially enrolled, more than half of them had to be excluded due to inferior image quality. This may partly be due to technical limitations of the transducer and 3DSTE but may also reflect patient-related factors such as lifestyle, body composition, smoking habits etc. Given the limited number of SCH patients, the study may be statistically underpowered to detect LA abnormalities; consequently, Type II errors remain a possibility despite high reproducibility. Therefore, it is important to emphasize that the presented findings need to be validated in larger, multi-center cohorts.
- Second, due to superior spatial and temporal resolution, 2D echocardiography maintains an advantage over 3DSTE in image quality. Additionally, the greater size of the 3DSTE probe complicates precise positioning. The multi-beat acquisition protocol (integrating six subvolumes) further introduces risks of stitching errors and motion artifacts, potentially compromising data integrity (14–17). These factors may have influenced the observed results and, consequently, the conclusions of the present study. Accordingly, further refinement of imaging protocols is essential to address these technical limitations and mitigate the aforementioned challenges in future research.
- Third, 3DSTE enables the simultaneous assessment of LV deformation and rotational mechanics, and valvular annular dimensions from the same dataset. Given that these cardiac chambers and valves are functionally interdependent, a comprehensive and concurrent evaluation of all components would be highly valuable; however, such a detailed analysis exceeded the predefined objectives of this investigation.
- Fourth, the present study did not aim to validate the calculated parameters, as their validity has already been established in previous studies (19,20).
- Fifth, the LA appendage and pulmonary veins were not included in the analysis. However, other morphological characteristics of the LA were examined (19,20).
- Sixth, although the anatomical attribution of the atrial septum remains debated, in this study it was considered as part of the LA.
- Finally, the cross-sectional design of this study precludes the establishment of causal relationships. Future longitudinal research would be essential to evaluate the progression of these LA abnormalities over time and to determine their correlation with clinical outcomes, such as cardiovascular events or the development of schizophrenic symptoms.

## 6. Conclusions

Significant volumetric and functional abnormalities of the left atrium could be detected in patients with treated chronic schizophrenia using three-dimensional speckle-tracking echocardiography.

## Figures and Tables

**Table 1:** The way to calculate left atrial stroke volumes and emptying fractions in each phasis of left atrial motion.

	Reservoir	Conduit Function	Active Contraction
**Stroke volumes** **(mL)**	Total SV = V_max_ − V_min_	Passive SV = V_max_ − V_preA_	Active SV = V_preA_ − V_min_
**Emptying fractions** **(%)**	Total EF = Total SV/V_max_	Passive EF = Passive SV/V_max_	Active EF = Active SV/V_preA_

Abbreviations: EF = emptying fraction, LV = left ventricular, SV: stroke volume, V_max_: maximum left atrial volume, V_min_: minimum left atrial volume, and V_preA_: left atrial volume before left atrial contraction.

**Table 2:** Two-dimensional echocardiographic parameters of patients with chronic schizophrenia and controls.

	Controls (n = 40)	Patients with Chronic Schizophrenia (n = 17)
LA diameter (mm)	37.3 ± 3.7	37.3 ± 2.9
LV end-diastolic diameter (mm)	48.5 ± 4.3	47.9 ± 3.4
LV end-diastolic volume (mL)	113.8 ± 35.7	107.4 ± 17.7
LV end-systolic diameter (mm)	32.6 ± 4.1	29.5 ± 2.0
LV end-systolic volume (mL)	39.9 ± 13.2	36.2 ± 5.9
Interventricular septum (mm)	9.3 ± 1.1	9.8 ± 0.9
LV posterior wall (mm)	9.4 ± 1.3	9.6 ± 0.7
LV ejection fraction (%)	64.6 ± 3.4	64.8 ± 3.6

Abbreviations: LA = left atrium and LV = left ventricle.

**Table 3:** Comparison of three-dimensional speckle-tracking echocardiography-derived volumetric left atrial parameters between patients with chronic schizophrenia and controls.

	Controls (n = 40)	Patients with Chronic Schizophrenia (n = 17)
**Left atrial volumes**		
V_min_ (mL)	21.8 ± 9.5	14.7 ± 2.5 *
V_preA_ (mL)	31.9 ± 12.1	21.1 ± 5.2 *
V_max_ (mL)	44.2 ± 13.7	32.7 ± 6.2 *
**Left atrial stroke volumes**		
TASV (mL)	22.4 ± 9.1	18.0 ± 5.4 *
PASV (mL)	12.3 ± 6.0	11.5 ± 5.0
AASV (mL)	10.1 ± 6.6	6.4 ± 4.3 *
**Left atrial emptying fractions**		
TAEF (%)	50.6 ± 14.3	54.2 ± 7.6
PAEF (%)	28.2 ± 12.2	35.0 ± 12.1 *
AAEF (%)	31.7 ± 13.2	28.3 ± 13.0

* *p* < 0.05 vs. controls. Abbreviations: V_max_ = maximum left atrial volume, V_min_ = minimum left atrial volume, V_preA_ = left atrial volume before left atrial contraction, TASV = total atrial stroke volume, TAEF = total atrial emptying fraction, AASV = active atrial stroke volume, AAEF = active atrial emptying fraction, PASV = passive atrial stroke volume, and PAEF = passive atrial emptying fraction.

**Table 4:** Comparison of three-dimensional speckle-tracking echocardiography-derived global and mean segmental peak left atrial strains in patients with chronic schizophrenia and controls.

	Controls (n = 40)	Patients with Chronic Schizophrenia (n = 17)
**Global strains**		
** Radial (%)**	−15.7 ± 10.5	−17.4 ± 8.9
** Circumferential (%)**	33.4 ± 15.7	37.9 ± 15.6
** Longitudinal (%)**	26.3 ± 10.0	28.6 ± 7.8
** 3D (%)**	−7.9 ± 7.1	−8.7 ± 7.0
** Area (%)**	65.0 ± 28.6	82.8 ± 32.8 *
**Mean segmental strains**		
** Radial (%)**	−19.6 ± 7.7	−23.8 ± 7.0 *
** Circumferential (%)**	37.2 ± 15.6	43.1 ± 15.7
** Longitudinal (%)**	29.5 ± 8.7	30.5 ± 8.2
** 3D (%)**	−13.0 ± 5.5	−15.6 ± 6.2
** Area (%)**	70.5 ± 28.7	90.3 ± 33.8 *

* *p* < 0.05 vs. controls. Abbreviations: 3D = three-dimensional.

**Table 5:** Comparison of three-dimensional speckle-tracking echocardiography-derived regional peak left atrial strains in patients with chronic schizophrenia and controls.

	Controls (n = 23)	Patients with Chronic Schizophrenia (n = 17)
**RS_basal_ (%)**	−19.3 ± 10.5	−20.6 ± 7.9
**RS_mid-atrial_ (%)**	−21.0 ± 8.9	−25.0 ± 8.0
**RS_superior_ (%)**	−20.1 ± 12.9	−26.9 ± 13.7
**CS_basal_ (%)**	43.2 ± 18.1	30.5 ± 10.3 *
**CS_mid-atrial_ (%)**	34.0 ± 14.6	35.7 ± 11.6
**CS_superior_ (%)**	37.2 ± 23.1	75.1 ± 41.5 *
**LS_basal_ (%)**	21.4 ± 10.5	21.7 ± 9.6
**LS_mid-atrial_ (%)**	39.2 ± 12.3	37.7 ± 13.5
**LS_superior_ (%)**	27.3 ± 14.6	36.9 ± 15.3 *
**3DS_basal_ (%)**	−13.7 ± 8.8	−14.4 ± 6.4
**3DS_mid-atrial_ (%)**	−12.6 ± 6.0	−15.7 ± 6.8
**3DS_superior_ (%)**	−13.6 ± 9.3	−17.3 ± 12.5
**AS_basal_ (%)**	62.7 ± 24.8	50.2 ± 16.5 *
**AS_mid-atrial_ (%)**	79.6 ± 31.6	80.7 ± 25.3
**AS_superior_ (%)**	75.9 ± 53.1	164.4 ± 116.9 *

* *p* < 0.05 vs. controls. Abbreviations: RS = radial strain, CS = circumferential strain, LS = longitudinal strain, 3DS = three-dimensional strain, and AS = area strain.

**Table 6:** Comparison of three-dimensional speckle-tracking echocardiography-derived global and mean segmental left atrial strains at atrial contraction in patients with chronic schizophrenia and controls.

	Controls (n = 40)	Patients with Chronic Schizophrenia (n = 17)
**Global strains**		
** Radial (%)**	−6.0 ± 5.5	−7.9 ± 5.0
** Circumferential (%)**	13.7 ± 10.4	18.1 ± 13.5
** Longitudinal (%)**	9.6 ± 6.9	12.0 ± 9.0
** 3D (%)**	−3.7 ± 4.5	−4.8 ± 5.8
** Area (%)**	25.7 ± 13.5	37.8 ± 25.1 *
**Mean segmental strains**		
** Radial (%)**	−8.4 ± 4.3	−12.4 ± 6.3 *
** Circumferential (%)**	15.8 ± 8.3	19.5 ± 10.5
** Longitudinal (%)**	9.3 ± 4.2	13.0 ± 5.7 *
** 3D (%)**	−5.8 ± 4.0	−7.3 ± 6.4
** Area (%)**	25.7 ± 13.5	37.5 ± 15.5 *

* *p* < 0.05 vs. controls. Abbreviations: 3D = three-dimensional.

**Table 7:** Comparison of three-dimensional speckle-tracking echocardiography-derived regional left atrial strains at atrial contraction in patients with chronic schizophrenia and controls.

	Controls (n = 40)	Patients with Chronic Schizophrenia (n = 17)
**RS_basal_ (%)**	−8.3 ± 5.1	−11.1 ± 6.2
**RS_mid-atrial_ (%)**	−8.8 ± 4.6	−12.8 ± 7.8 *
**RS_superior_ (%)**	−8.0 ± 7.6	−13.9 ± 10.5 *
**CS_basal_ (%)**	18.0 ± 9.1	16.9 ± 9.3
**CS_mid-atrial_ (%)**	13.1 ± 8.8	16.5 ± 9.3
**CS_superior_ (%)**	14.9 ± 11.1	34.2 ± 28.0 *
**LS_basal_ (%)**	6.8 ± 4.2	9.6 ± 5.7 *
**LS_mid-atrial_ (%)**	10.9 ± 7.1	14.7 ± 8.8
**LS_superior_ (%)**	10.8 ± 7.0	18.6 ± 12.1 *
**3DS_basal_ (%)**	−6.5 ± 5.8	−7.9 ± 5.1
**3DS_mid-atrial_ (%)**	−5.6 ± 3.5	−8.5 ± 6.3 *
**3DS_superior_ (%)**	−5.0 ± 7.5	−8.3 ± 8.1
**AS_basal_ (%)**	24.1 ± 13.2	24.2 ± 12.3
**AS_mid-atrial_ (%)**	26.4 ± 15.9	36.3 ± 22.4 *
**AS_superior_ (%)**	27.1 ± 24.0	70.3 ± 55.4 *

* *p* < 0.05 vs. controls. Abbreviations: RS = radial strain; CS = circumferential strain; LS = longitudinal strain; 3DS = three-dimensional strain; AS = area strain.

## Data Availability

The data presented in this study are available on request from the corresponding author.
